# Defects in MAP1S‐mediated autophagy cause reduction in mouse lifespans especially when fibronectin is overexpressed

**DOI:** 10.1111/acel.12441

**Published:** 2016-01-10

**Authors:** Wenjiao Li, Jing Zou, Fei Yue, Kun Song, Qi Chen, Wallace L. McKeehan, Fen Wang, Guibin Xu, Hai Huang, Jinglin Yi, Leyuan Liu

**Affiliations:** ^1^ Center for Translational Cancer ResearchInstitute of Biosciences and TechnologyTexas A&M Health Science Center2121 W. Holcombe Blvd.HoustonTX77030USA; ^2^ Department of ophthalmologyXiangya HospitalCentral South UniversityChangshaHunan410008China; ^3^ Jiangxi Research Institute of Ophthalmology and Visual SciencesThe Affiliated Eye Hospital of Nanchang UniversityNanchang330006China; ^4^ Department of UrologyThe Sun Yat‐sen Memorial HospitalSun Yat‐sen UniversityGuangzhou510120China; ^5^ Department of Molecular and Cellular MedicineCollege of MedicineTexas A&M Health Science CenterCollege StationTX77843USA

**Keywords:** autophagy, fibronectin, lifespan, liver fibrosis, MAP1S

## Abstract

Autophagy is a cellular process that executes the turnover of dysfunctional organelles and misfolded or abnormally aggregated proteins. Microtubule‐associated protein MAP1S interacts with autophagy marker LC3 and positively regulates autophagy flux. LC3 binds with *fibronectin* mRNA and facilitates its translation. The synthesized fibronectin protein is exported to cell surface to initiate the assembly of fibronectin extracellular matrix. Fibronectin is degraded in lysosomes after it is engulfed into cytosol via endocytosis. Here, we show that defects in MAP1S‐mediated autophagy trigger oxidative stress, sinusoidal dilation, and lifespan reduction. Overexpression of LC3 in wild‐type mice increases the levels of fibronectin and γ‐H_2_ AX, a marker of DNA double‐strand breakage. LC3‐induced fibronectin is efficiently degraded in lysosomes to maintain a balance of fibronectin levels in wild‐type mice so that the mice live a normal term of lifespan. In the LC3 transgenic mice with MAP1S deleted, LC3 enhances the synthesis of fibronectin but the MAP1S depletion causes an impairment of the lysosomal degradation of fibronectin. The accumulation of fibronectin protein promotes liver fibrosis, induces an accumulation of cell population at the G0/G1 stage, and further intensifies oxidative stress and sinusoidal dilatation. The LC3‐induced overexpression of fibronectin imposes stresses on MAP1S‐deficient mice and dramatically reduces their lifespans. Therefore, MAP1S‐mediated autophagy plays an important role in maintaining mouse lifespan especially in the presence of extra amount of fibronectin.

## Introduction

Mammalian cells primarily use the autophagy–lysosome pathway to degrade dysfunctional organelles, misfolded/aggregated proteins, and other macromolecules (Mizushima *et al*., 1998). Autophagy defects lead to enhancement of oxidative stresses (Mizushima *et al*., 1998; Liu *et al*., 2012). Oxidative stress in turn activates NLRP3 inflammasome and eventually induces an inflammatory form of cell death referred to as pyroptosis that promotes the mortality and impair the survival of host structural, hematopoietic, and immune‐competent cells (Lamkanfi & Dixit, 2014; Ryter *et al*., 2014; Terlizzi *et al*., 2014; Yu *et al*., 2014). Cancer patients with defective autophagy live a short lifespans (Jiang *et al*., 2014, 2015). Both calorie restriction and pharmacologic agents that mimic the impact of calorie restriction induce autophagy and prolong lifespans (Madeo *et al*., 2015). Therefore, autophagy plays an important role in longevity.

**Figure 1:**
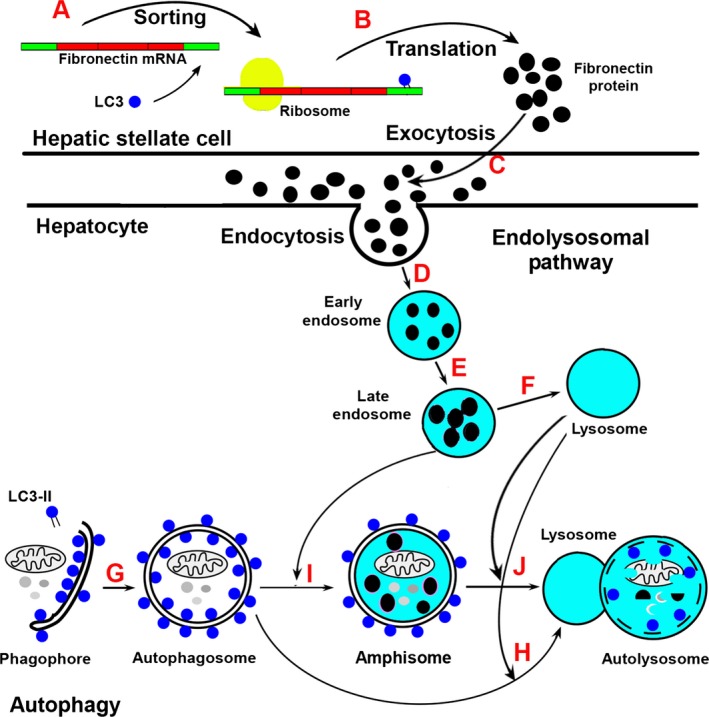
LC3 is involved in the regulation of fibronectin biosynthesis and autophagy flux. (A, B) LC3 binds with *fibronectin* mRNA to facilitate its sorting to ribosome (A) where it is translated to fibronectin protein (B). (C) Fibronectin is secreted into extracellular surface through exocytosis. (D–F) Fibronectin enters cytosol through endocytosis to form early endosome (C), then late endosome (E) that directly fuses with lysosome to be degraded (F). (G, H) LC3‐II binds with phagophore to form autophagosome (G) and fuses with lysosome to form autolysosome (H). (I, J) Late endosome fuses with autophagosome to form amphisome (I) and then fuses with lysosome to form autolysosome (J). This schematic drawing is modified from the Fig. 1 published by Hansen & Johansen (2011).

LC3 was originally discovered as an interactive protein of the microtubule‐associated protein MAP1A, MAP1B, and MAP1S (Schoenfeld *et al*., 1989; Xie *et al*., 2011a). LC3 binds with *fibronectin* mRNA and facilitates the sorting of *fibronectin* mRNA along microtubules onto rough endoplasmic reticulum where the mRNA is translated (Fig. 1A,B) (Zhou & Rabinovitch, 1998). After being translated and exported to the surface of plasma membrane through exocytosis (Fig. 1C) (Lobert *et al*., 2010), fibronectin initiates the assembly of fibronectin extracellular matrix and other extracellular matrix proteins such as Collagen (Singh *et al*., 2010). Following endocytosis, fibronectin is packaged into early endosome, matured to late endosome, and directly degraded in lysosomes (Fig. 1D–F) (Hansen & Johansen, 2011). In response to liver damage, hepatic stellate cells become activated to enhance the production of cellular fibronectin while the clearance of cellular fibronectin by hepatocytes is impaired so that more fibronectins are accumulated. The accumulation of fibronectin further leads to an activation of the activity of hepatic stellate cells to mediate extracellular matrix remodeling and generate matrix protein constituents in the connective scar tissue (Moreira, 2007; Zhang & Friedman, 2012).

Interestingly, LC3 is one of the key autophagy markers in mammalian cells. LC3 precursor is truncated to form the cytosolic LC3‐I and further conjugated with phosphatidylethanolamine to create the membrane‐associated LC3‐II (Tanida *et al*., 2004). The LC3‐II‐associated isolation membranes target to and then completely envelop organelles or macromolecules to form autophagosomes (Fig. 1G). Autophagosomes migrate along acetylated microtubules to fuse with lysosomes to form autolysosomes in which substrates are degraded (Fig. 1H) (Mizushima *et al*., 2008; Xie *et al*., 2010). Fibronectin‐containing late endosome may also fuses with autophagosome to form amphisome, and then fuses with lysosome to form autolysosomes (Fig. 1I,J) (Hansen & Johansen, 2011). MAP1S interacts with both LC3‐I and LC3‐II and positively regulates autophagosomal biogenesis and degradation autophagy by bridging autophagic components with microtubules (Xie *et al*., 2011a). Thus, LC3 regulates not only synthesis of fibronectin but also autophagy.

Possibly due to isoform redundancy, LC3 beta knockout mice maintain the same levels of fibronectin protein as the wild‐type mice, exhibit no defect in autophagy, and develop normally (Cann *et al*., 2008). Because of the interaction between MAP1S and LC3 and the association of LC3 with both fibronectin and autophagy, we were triggered to study the impact of LC3‐induced overexpression of fibronectin on the lifespan of the MAP1S‐deficient mice with a defective autophagy. Here, we show that defects in MAP1S‐mediated autophagy trigger oxidative stress, sinusoidal dilatation, and lifespan reduction in mice. Overexpression of LC3 in wild‐type mice promotes not only the synthesis but also the degradation of fibronectin, so a balance in levels of fibronectin is maintained. Thus, the LC3 transgenic mice have a normal lifespan as the wild‐type mice and were routinely adopted as a model for *in vivo* autophagy analysis (Kuma & Mizushima, 2007). In the LC3 transgenic MAP1S‐deficient mice, LC3 overexpression enhance the synthesis, but the MAP1S deficiency reduced the efficiency of lysosomal degradation of fibronectin. The resulted accumulation of fibronectin protein in liver tissues triggers liver fibrosis and cell cycle arrest, and dramatically reduces the lifespan that has already been shortened by MAP1S deletion.

## Results

### LC3 overexpression causes accumulation of fibronectin protein

**Figure 2:**
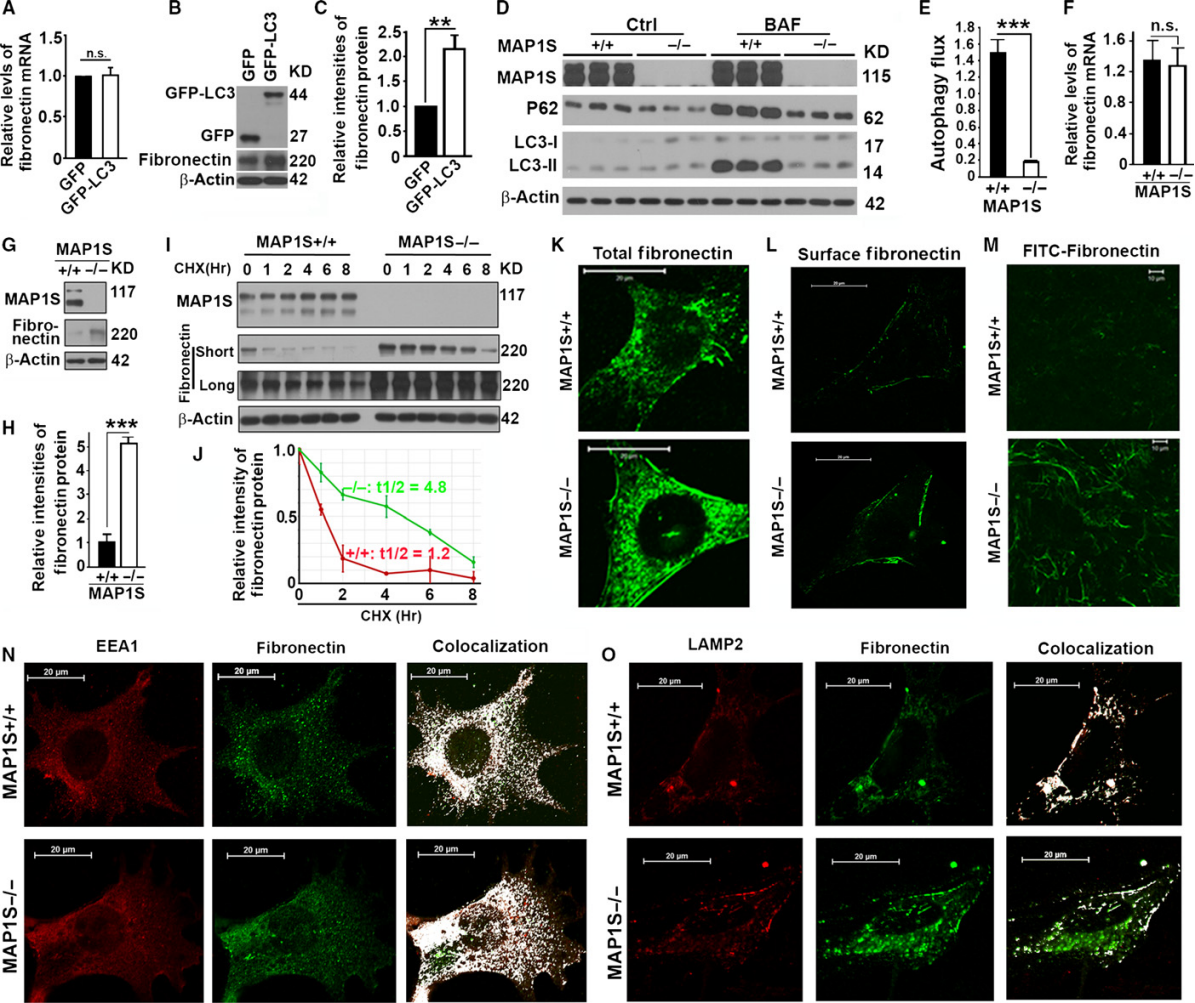
LC3 overexpression causes accumulation of fibronectin protein, but MAP1S enhances turnover of fibronectin in lysosomes. (A) A plot of relative levels of *fibronectin* mRNA in MEFs transiently expressing GFP and GFP‐LC3. (B) A representative immunoblot of lysates from the same cells as described in (A). Lysates from wild‐type MEFs transiently expressing GFP or GFP‐LC3 with the same amount of total proteins were subjected to immunoblot with antibody against GFP, fibronectin, or β‐actin. (C) A plot of relative intensities of fibronectin revealed by immunoblot shown in (B). (D) Immunoblot analyses of lysates of MEFs developed from MAP1S^+/+^ and MAP1S^−/−^ mice. Cells were cultured in the absence or presence of 10 nm bafilomycin A1 (BAF) for 6 hrs. Lysates with the same amount of total proteins from three experiments were subjected to immunoblot with antibody against LC3 or β‐actin on the same gels. (E) A plot of autophagy flux as indicated by the increased levels of LC3‐II after bafilomycin A1 treatment as shown in (D). (F) A plot of relative levels of *fibronectin* mRNA in MEFs developed from MAP1S^+/+^ or MAP1S^−/−^ mice. (G) A representative immunoblot of lysates from the same cells as described in (F). Lysates with the same amount of total proteins were subjected to immunoblot with antibody against MAP1S, fibronectin, or β‐actin. (H) A plot of relative intensities of fibronectin revealed by immunoblot shown in (G). (I) Analyses of the impact of MAP1S on the stability of fibronectin protein. MEFs were grown to confluent and protein translation was inhibited with cycloheximide (CHX). Cell lysates were collected at different time points, and the same amounts of total proteins were loaded and β‐actin served as another loading control. Lysates with the same amount of total proteins were subjected to immunoblot with antibody against MAP1S, fibronectin, or β‐actin. ‘Short’ or ‘long’ indicates the exposure times for immunoblotting selected to show a linear relation of signal and protein concentration. (J) Plots of relative intensities of fibronectin as shown in (I). The initial intensities of fibronectin at time zero in two different types of MEFs were set to be 1. (K) Immunostaining analyses of fibronectin in MEFs using antifibronectin antibody. (L) Immunostaining analyses of fibronectin on the cell surface of MEFs using antifibronectin antibody. Cells were immunostained similarly as in (K) except that cells were not treated with Triton‐100 to permeate the cell membranes. (M) Fluorescent imaging of MEFs incubated with FITC‐conjugated fibronectin (FITC‐fibronectin). (N) A colocalization analysis of fibronectin with EEA1‐labeled early endosomes in wild‐type and MAP1S^−/−^ MEFs. White signals in the panel of Colocalization indicates overlapping between EEA1 and fibronectin as analyzed by ImageJ software. (O) A colocalization analysis of fibronectin with LAMP2‐labeled lysosomes in wild‐type and MAP1S^−/−^ MEFs stained with antibodies against LAMP2 and fibronectin. Colocalization analysis was performed similarly as in (N). Data shown in plots above were the averages and standard deviations of three repeats. The significances of differences were determined by Student's *t*‐test. n.s., not significant, **, and ***, *P *≤ 0.001.

LC3 was reported to bind with *fibronectin* mRNA to enhance the efficiency of translation to fibronectin protein (Zhou *et al*., 1997). As expected, overexpression of LC3 in MEFs did not alter the levels of *fibronectin* mRNA (Fig. 2A) but significantly increased the levels of fibronectin protein (Fig. 2B,C). Thus, overexpression of LC3 causes the accumulation of fibronectin.

### MAP1S deficiency leads to an impairment of autophagy flux

Although a reduction in levels of Bcl‐2 and Bcl‐XL may suggest an activation of autophagy initiation (Pattingre *et al*., 2005), the simultaneous reduction in levels of P27 in addition to Bcl‐2 and Bcl‐XL indicates an impaired autophagy flux (Liang *et al*., 2007). MAP1S enhances autophagy initiation through the LKB1‐AMPK‐mTOR pathway by sustaining the levels of Bcl‐2/XL and P27 (Xie *et al*., 2011a; Liu *et al*., 2012). We cultured MEFs developed from wild‐type and MAP1S^−/−^ mice in the absence or presence of lysosomal inhibitor bafilomycin A1, and found that autophagy flux indicated by the increased levels of LC3‐II after treatment with bafilomycin A1 for the same time was dramatically reduced by the MAP1S depletion (Fig. 2D,E). The results further confirm that MAP1S is a positive regulator of autophagy flux.

### MAP1S facilitates the endocytosis and lysosomal turnover of fibronectin

We found no difference in the levels of *fibronectin* mRNA between the wild‐type and MAP1S^−/−^ MEFs (Fig. 2F). MAP1S deletion caused a significant increase in the levels of fibronectin protein (Fig. 2G,H). The stability of protein is measured by T1/2, the time for the protein to be degraded to reach a half of the total protein after protein synthesis is terminated by cycloheximide. MAP1S deletion increased the stability of fibronectin from 1.2 to 4.8 h (Fig. 2I,J). MAP1S^−/−^ MEFs accumulated higher levels of fibronectin protein both inside and outside of cells than the wild type (Fig. 2K,L). Accumulation of intracellular fibronectin in the MAP1S^−/−^ MEFs indicated that the efficiency of lysosomal turnover and/or the secretion of fibronectin was impaired, while an accumulation of more surface fibronectin suggested that the secretion of fibronectin likely functioned normally but cellular uptake of surface fibronectin was impaired. To further understand the mechanism, we performed an absorption assay by incubating MEFs with exogenous FITC‐fibronectin. MEFs were incubated with the same amounts of FITC‐labeled purified fibronectin for overnight and then washed with fresh medium. Wild‐type MEFs efficiently absorbed the FITC‐fibronectin into cytosol and degraded it. In MAP1S^−/−^ MEFs, more FITC‐fibronectin accumulated on the cell surface and in the cytosol (Fig. 2M). Fibronectin was reported to be engulfed in endosomes and degraded in lysosomes (Lobert *et al*., 2010). Closer examination revealed that endogenous fibronectin was colocalized with EEA1‐labeled early endosomes (Fig. 2N) and LAMP2‐labeled lysosomes (Fig. 2O). Most of cytosolic fibronectin punctate foci in both wild‐type and MAP1S^−/−^ MEFs were colocalized with EEA1 (Fig. 2N), while more cytosolic fibronectin punctate foci in MAP1S^−/−^ MEFs were not colocalized with LAMP2‐labeled lysosomes than in the wild type (Fig. 2O). Therefore, MAP1S facilitates the lysosomal turnover of fibronectin.

### LC3 overexpression and MAP1S deletion synergistically cause an accumulation of fibronectin, TGF‐β, and α‐SMA, and induce liver fibrosis

**Figure 3:**
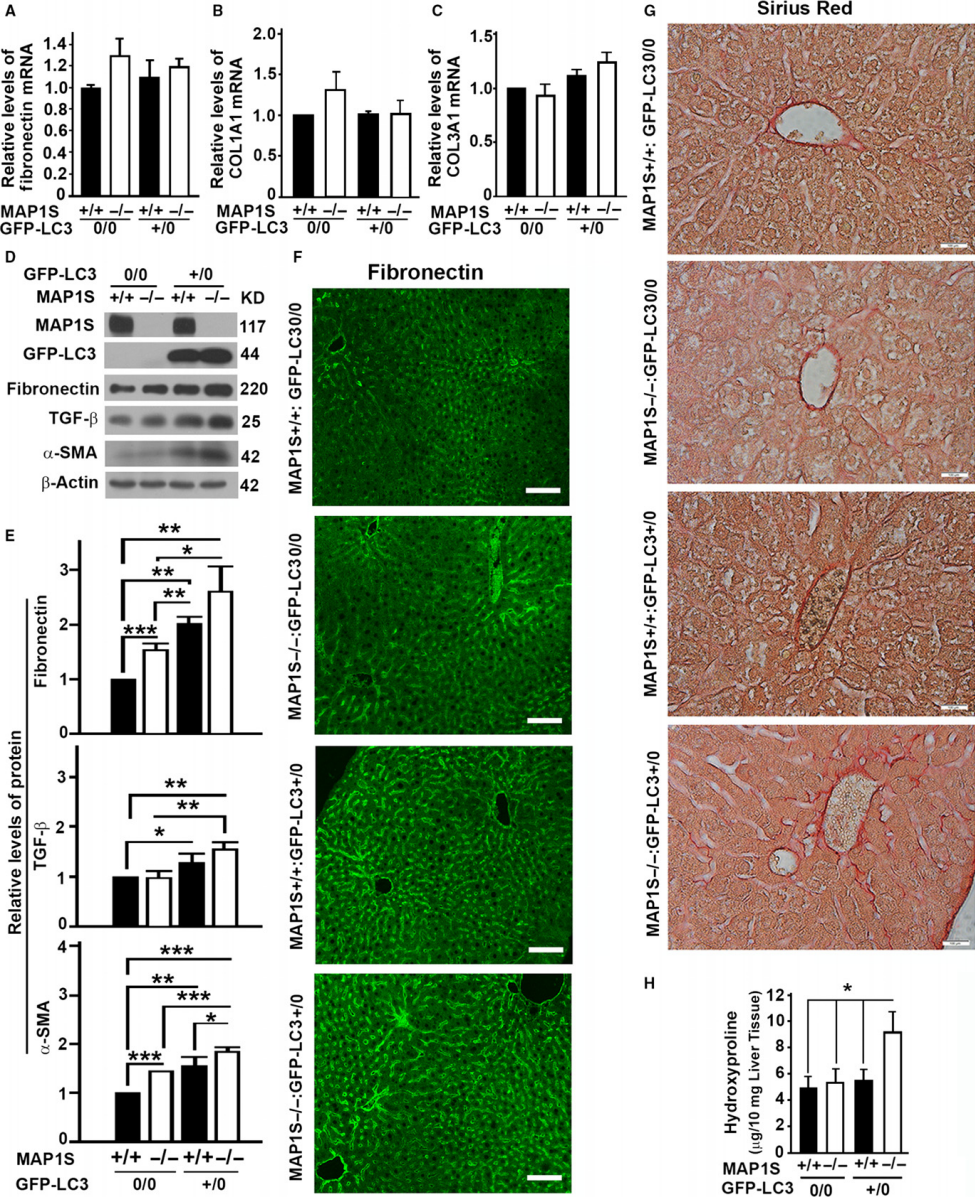
LC3 overexpression and MAP1S deletion synergistically causes accumulation of fibronectin, TGF‐β and α‐SMA, and development of liver fibrosis. (A–C) Plots of relative levels of *fibronectin* (A), *Collagen, Type I, Alpha 1 (COL1A1)* (B) and *Collagen, Type III, Alpha 1 (COL3A1)* mRNAs (C) in mouse liver tissues from four different genotypes at 6 months of age. (D) A representative immunoblot of lysates from the same liver tissues as described in (A). Lysates with the same amount of total proteins were subjected to immunoblot with antibody against MAP1S, GFP, fibronectin, TGF‐β, α‐SMA, or β‐actin. (E) A plot of relative intensities of fibronectin, TGF‐β, and α‐SMA in liver tissues from mice as shown as representative in (D). The initial intensity of each protein in the wild type was set to be 1. Data shown in plots above were the averages and standard deviations of three repeats. (F) Immunostaining analyses of fibronectin in sections from liver tissues described in (A) using antifibronectin antibody. Bar: 100 μm. (G) Comparative Sirius Red staining among the livers of the four different genotypes. Bar = 100 μm. (H) Plots of the levels of hydroxyproline in liver tissues of the four different genotypes. Plots were the means ± SD of three repeats, and the significance of the differences was compared using Student's *t*‐test. *, *P *≤ 0.05; **, *P *≤ 0.01; and ***, *P *≤ 0.001.

MAP1S knockout mice were founded to be normal until 12 months old (Xie *et al*., 2011a,b). GFP‐LC3 transgenic mice have been widely used as models for autophagy research (Kuma & Mizushima, 2007). To study the impact of MAP1S on LC3‐induced overexpression of fibronectin, the MAP1S knockout mice and the GFP‐LC3 transgenic mice were crossed to produce mice with the same genetic background but with four different genotypes: 1) the wild‐type mice (MAP1S^+/+^:GFP‐LC3^0/0^), 2) MAP1S knockout mice (MAP1S^−/−^:GFP‐LC3^0/0^), 3) transgenic mice carrying a single copy of GFP‐LC3 (MAP1S^+/+^:GFP‐LC3^+/0^), and 4) GFP‐LC3 transgenic MAP1S knockout mice (MAP1S^−/−^:GFP‐LC3^+/0^). There was no difference in levels of *fibronectin* and *collagen* mRNAs among liver tissues from mice of four different genotypes (Fig. 3A–C). However, a similar LC3 overexpression‐driven increase in the levels of fibronectin protein was observed in both MAP1S^+/+^ and MAP1S^−/−^ mice (Fig. 3D,E). The accumulated fibronectin mainly distributed in the sinusoidal space of liver tissues (Fig. 3F). Other fibrosis‐related proteins, TGF‐β and α‐smooth muscle actin (α‐SMA), were also increased along with the LC3 overexpression in 6‐month‐old mice (Fig. 3D,E). Only the MAP1S^−/−^:GFP‐LC3^+/0^ mice developed liver fibrosis as indicated by Sirius Red staining (Fig. 3G) and levels of hydroxyproline (Fig. 3H). Therefore, LC3‐induced overexpression of fibronectin leads to development of liver fibrosis in autophagy‐defective mice.

### LC3 overexpression and MAP1S deletion synergistically alter the cell populations at different stages of cell cycle

**Figure 4:**
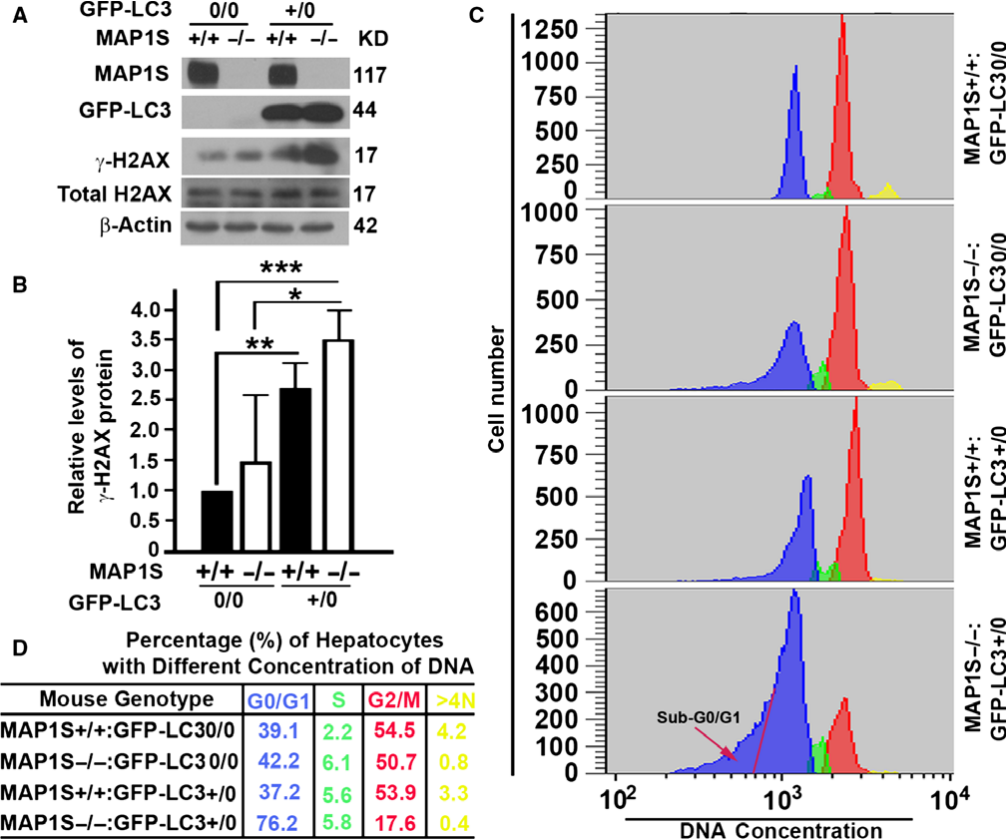
LC3 overexpression and MAP1S deletion synergistically suppress the progress of cell cycle. (A) A representative immunoblot of liver lysates from four different genotypes at 6 months of age. Lysates with the same amount of total proteins were subjected to immunoblot with antibody against MAP1S, GFP, γ‐H2AX, H2AX, or β‐actin. (B) Plots of relative intensities of γ‐H2AX bands in the liver lysates from four different genotypes. The value in wild‐type mouse was set as standard 1. Data are the average and standard deviation of three mice. The significances of differences between wild‐type and knockout mice were determined by Student's *t*‐test. *, *P *≤ 0.05; **, *P *≤ 0.01; and ***, *P *≤ 0.001. (C) Flow cytometric analyses of hepatic nuclei isolated from liver tissues of four different genotypes of mice. Mice were subjected to analysis for their DNA contents by propidium iodide (PI) staining, and their histograms in different colors were aligned with each other. The red arrow points to the cell population with sub‐G0/G1 DNA concentration. (D) A table summarizing the percentages of hepatocytes with nuclear DNA contents around peaks of G0/G1, S, G2/M, or >4N to the total number of hepatocytes in each sample were summarized.

The γ‐H2AX‐labeled DNA double‐strand breaks in mouse liver were considered as a marker of aging (Sedelnikova *et al*., 2004). Although the levels of total H2AX remained constant among four different types of mice, GFP‐LC3 transgenic mice always had higher levels γ‐H2AX protein than the nontransgenic counterparts in both the wild‐type and the MAP1S^−/−^ mice, but the deletion of MAP1S exhibited no dramatic impact on the levels of γ‐H2AX (Fig. 4A,B). The deletion of MAP1S also exhibited no dramatic impact on the DNA concentration in liver, and the high levels of γ‐H2AX protein induced by LC3 overexpression did not alter the profiles of DNA concentrations in the liver tissues of wild‐type mice. However, the LC3 overexpression triggered a dramatic increase of cell population in G0/G1 but a dramatic reduction in the cell population in G2/M stage of cell cycle in the liver of MAP1S^−/−^ mice (Fig. 4C,D). Meanwhile, increasing levels of aneuploidy cells represented by the sub‐G0/G1 population were observed in LC3 transgenic MAP1S^−/−^ mice (Fig. 4C). Therefore, LC3 overexpression and MAP1S deletion synergistically induce genome instability and alter the cell populations at different stages of cell cycle.

### LC3 overexpression exacerbates MAP1S deficiency‐induced autophagy defects and oxidative stress

**Figure 5:**
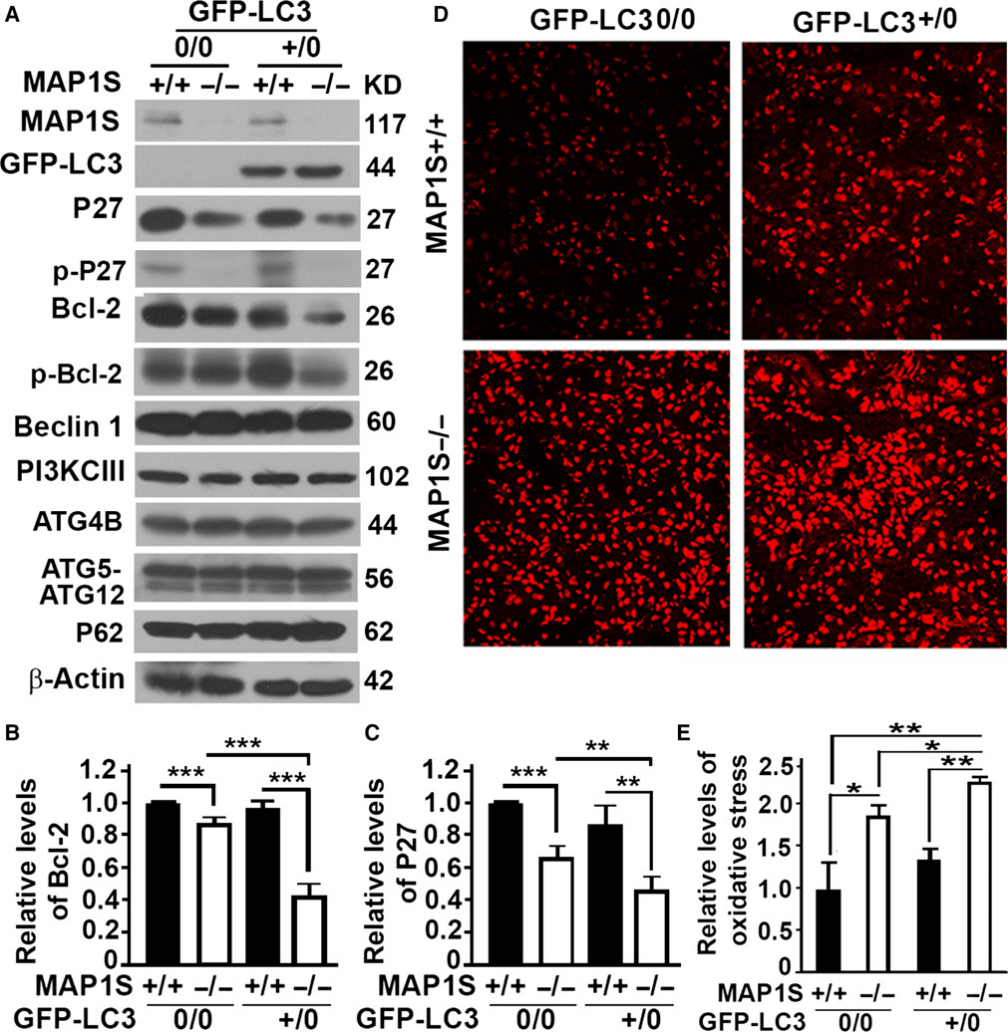
LC3 overexpression exacerbates MAP1S deletion‐induced autophagy defects and oxidative stress. (A–C) Representative immunoblot (A) and quantification of the relative levels of Bcl‐2 (B) and P27 (C) of liver lysates from four different genotypes at 12 months of age. Lysates with the same amount of total proteins were subjected to immunoblot with antibody against MAP1S, GFP, P27, phosphorylated P27 (p‐P27), Bcl‐2, phosphorylated Bcl‐2 (p‐Bcl‐2), Beclin 1, PI3KCIII, ATG4B, ATG5, P62, or β‐actin. (D) Comparative analyses of the levels of oxidative stress among the livers of the four different genotypes by staining with dihydroethidine hydrochloride. Bar = 100 μm. (E) Plots of the relative levels of oxidative stress of the four different genotypes. Plots were the means ± SD of three mice, and the significance of the differences was compared using Student's *t*‐test. *, *P *< 0.05. **, *P *≤ 0.01 and ***, *P *≤ 0.001.

As we previously reported, MAP1S depletion had no impact on proteins involved in the regulation of autophagy initiation such as Beclin 1, PI3KCIII, ATG4B, and ATG5, but led to decreases in both Bcl‐2 and P27 levels (Fig. 5A–C), suggesting an impairment of autophagy initiation through the LKB1‐AMPK‐mTOR pathway (Xie *et al*., 2011a). The levels of P62 remained constant among liver tissues from four different types of mice (Fig. 5A). The levels of both Bcl‐2 and P27 were further decreased in MAP1S^−/−^ mice expressing GFP‐LC3 (Fig. 5A–C), indicating that LC3 overexpression caused a further impairment of autophagy initiation. Autophagy defects lead to an enhancement of oxidative stresses (Mizushima *et al*., 1998; Liu *et al*., 2012). As expected, the MAP1S^−/−^ mice exhibited significantly higher levels of oxidative stress in liver tissues as indicated by the intensity of staining with dihydroethidine hydrochloride in nuclei than the wild‐type mice. LC3 overexpression further intensified the oxidative stress in the liver tissues of MAP1S^−/−^ mice (Fig. 5D,E).

### LC3 enhances MAP1S deletion‐induced sinusoidal dilatation

**Figure 6:**
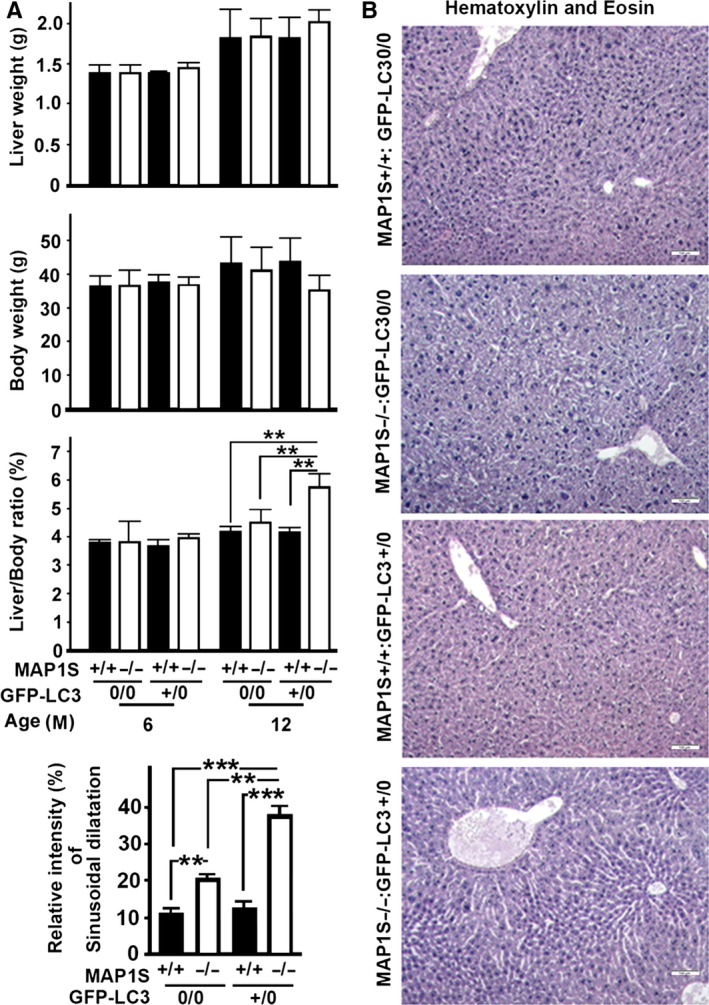
LC3 overexpression and MAP1S deletion synergistically promote sinusoidal dilatation. (A) Plots of liver weight, body weight, and ratio of liver weight to body weight of the four different genotypes at different ages. Plots were the means ± SD of three mice, and the significance of the differences was compared using Student's *t*‐test. **, *P *≤ 0.01. (B) Comparative hematoxylin and eosin staining among the liver tissues from four different genotypes. Bar = 100 μm. (C) Plots of the relative intensities of sinusoidal dilatation of the four different genotypes. Plots were the means ± SD of three mice, and the significance of the differences was compared using Student's *t*‐test. **, *P *≤ 0.01 and ***, *P *≤ 0.001.

Although the liver weights and body weights were not significantly different among different genotypes, there were significant differences in the ratios of liver weights to body weights among different types of mice at age of 12 months. The MAP1S^−/−^:GFP‐LC3^+/0^ mice exhibited the highest ratio (Fig. 6A). Histological examination revealed that MAP1S deletion caused sinusoidal dilatation, and LC3 overexpression had no significant impact on sinusoidal dilatation alone but dramatically amplified the intensities of sinusoidal dilatation triggered by MAP1S deletion (Fig. 6B,C).

### LC3 exacerbates the reduction in mouse lifespan caused by MAP1S deletion

**Figure 7:**
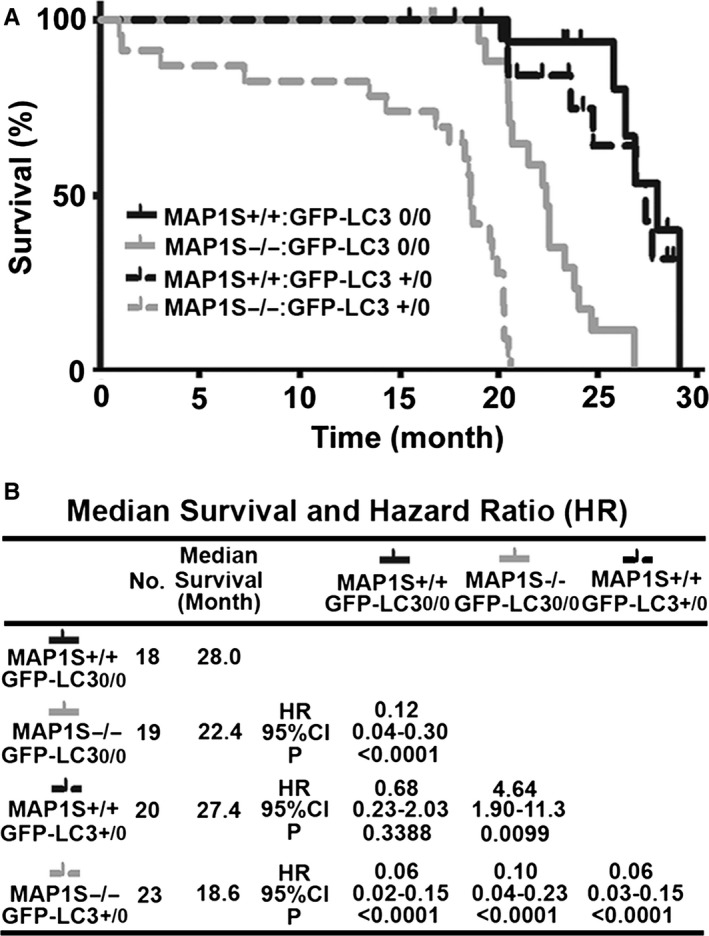
LC3 overexpression promotes the reduction in mouse lifespans caused by MAP1S deletion. (A) The Kaplan–Meier survival curves showing the survival time of four different genotypes with male and female mice in combination. (B) A table summarizes median survival and hazard ratio based on the plots in (B). No., number of mice; HR, hazard ratio; and 95% CI, 95% confidence interval. The significance of difference between two groups was estimated by log‐rank test, and *P* value for each plot was the probability larger than the chi‐square value.

Mice expressing an extra copy of GFP‐LC3 lived a normal lifespan as the wild‐type mice did. MAP1S deletion caused a significant 5.6‐month reduction in mouse lifespan (or 20% reduction in median survival, hazard ratio of 0.12). A combination of MAP1S deletion with GFP‐LC3 overexpression led to a synergistic 9.4‐month reduction in mouse lifespan (or 33% reduction in median survival, hazard ratio of 0.06) (Fig. 7). Therefore, LC3 overexpression exacerbates the reduction in mouse lifespan triggered by MAP1S deletion.

## Discussion

Fibronectins include two major forms: the hepatocyte‐produced plasma fibronectin cycling in blood and cellular fibronectin locally produced in different tissues by their resident fibroblasts and endothelial cells (Aziz‐Seible & Casey, 2011). In liver tissues, the fibronectin deposited in the pericellular matrix is synthesized by sinusoidal endothelial cells and hepatic stellate cells and degraded in the lysosomes of hepatocytes and other cell types via the endosomal sorting complex required for transport (ESCRT) machinery when fibronectin becomes damaged (Lobert *et al*., 2010; Aziz‐Seible & Casey, 2011). Under normal physiological conditions, there is a balance of both types of fibronectin in intact tissues (Aziz‐Seible & Casey, 2011). LC3 knockout mice exhibit a reduced synthesis of fibronectin but maintain the same levels of fibronectin protein as in the wild type and develop normally, suggesting unknown compensatory mechanisms for loss of LC3 to ensure a proper accumulation of fibronectin (Cann *et al*., 2008). Our results indicated that overexpression of LC3 led to increases in the synthesis of fibronectin protein. Wild‐type mice retained a sufficient autophagy flux to clean up the increased amount of damaged fibronectin caused by the increased amount of total synthesized fibronectin. MAP1S‐deficient mice had a reduced lysosomal capacity to deal with the endogenous damaged fibronectin, so an accumulation of fibronectin was observed. When MAP1S was deleted in GFP‐LC3 transgenic mice, the mice became overloaded with fibronectin and accumulated more fibronectin in the sinusoidal space of liver tissues because of the synergistic outcomes of enhanced synthesis and impaired degradation. Active TGF‐β is a pro‐fibrotic protein (Leask & Abraham, 2004). Fibronectin‐organized extracellular matrix in the sinusoidal space sequesters the latent TGF‐β (Wipff & Hinz, 2008). The resulted imbalance between fibronectin and TGF‐β (Wight & Potter‐Perigo, 2011) causes an activation of hepatic stellate cells (Aziz‐Seible *et al*., 2011) and development of liver fibrosis (Wight & Potter‐Perigo, 2011). Therefore, LC3‐induced overexpression of fibronectin directly leads to liver fibrosis when autophagy flux is blocked.

It is obvious that overexpression of GFP‐LC3 leads to increases in levels of both fibronectin and γ‐H_2_AX in both wild‐type and MAP1S^−/−^ mice. A positive collaboration between fibronectin and γ‐H_2_AX was previously reported in human colon cancer cells. Fibronectin may promote DNA double‐strand breakage in an α5 integrin and cell cycle‐dependent manner (De Wever *et al*., 2011). However, the exact mechanism by which fibronectin promotes DNA double‐strand breakage is not well understood. High levels of fibronectin and γ‐H_2_AX may enhance oxidative stress in liver tissues but exert no significant impact on the lifespan of the GFP‐LC3 transgenic MAP1S^+/+^ mice. Therefore, the level of γ‐H_2_AX is not a reliable marker of aging.

Our results indicate that short mouse lifespans are correlated with weak autophagy activity, high levels of oxidative stress, and high degree of sinusoidal dilation. Both Bcl‐2 and P27 are not only involved in the regulation of autophagy initiation but also in other functions. P27 was suggested to inhibit cell cycle and arrest cells at G0/G1 phase (Chen *et al*., 2008). Logically, a reduction in P27 levels would lead to an accumulation of cell population at G2/M phase, which is contradictory to our results shown in Fig. 4C,D. We reason that loss of P27 caused more cells to enter M/G2 phase as predicted (Chen *et al*., 2008); the high frequency of DNA double‐strand breaks caused by LC3 overexpression induced a prolonged arrest of cells at mitotic stage and eventually mitotic cell death as we previously reported (Liu *et al*., 2009); and, after a balance is reached, the cell population at G0/G1 phase was increased due to the reduction in cell population at G2/M phase in the MAP1S^−/−^: GFP‐LC3^0/+^ mice. It may also similarly explain the decrease in polyploidy cells in MAP1S^−/−^ mice irrespective of LC3 overexpression. Autophagy defects lead to enhancement of oxidative stresses and promotion of inflammation and pyroptosis (Mizushima *et al*., 1998; Liu *et al*., 2012; Lamkanfi & Dixit, 2014). Sinusoidal dilatation is a type of vascular liver lesions previously reported to be associated with inflammatory hepatocellular adenoma (formerly known as telangiectatic focal nodular hyperplasia) (Laumonier *et al*., 2008). It is characterized by widening of hepatic capillaries and may impair their contractile properties (Saadoun *et al*., 2004). The sinusoidal dilatation displayed in the liver tissues of MAP1S^−/−^ and MAP1S^−/−^:GFP‐LC3^+/0^ mice may induce dysfunction in the contractility of hepatic capillaries and liver failure. In addition, a general deletion of MAP1S in whole body of mice may also cause the dysfunction and failure of multiple organs and trigger an early termination of lifespan.

Further overexpression of LC3 in MAP1S^−/−^ mice induced higher stress of fibronectin deposition and led to obvious liver fibrosis. In addition, increased deposition of fibronectin not only causes hepatic fibrosis and sinusoidal dilatation but also contributes to a number of other pathological conditions such as pulmonary fibrosis, diabetic nephropathy, retinopathy, and macroangiopathy (Labat‐Robert, 2002). Fibronectin deposition in the sinusoidal space also impacts the adjacent hepatocytes. A strong cooperation between hepatocytes and mesenchymal cells and between the different nonparenchymal cell types occurs during liver injury (Gressner & Bachem, 1994). Meanwhile, the aneuploidy cells which appear in the sub‐G0/G1 population in our cell sorting analysis inherently trigger cell death (Liu *et al*., 2012; Silk *et al*., 2013). The increasing number of aneuploidy hepatocytes suggests an enhanced death of hepatocytes and consequently liver dysfunction and termination of mouse lifespan. Therefore, the autophagy‐defective mice exhibit the shortest lifespan under the stress of an overdeposition of fibronectin.

Although overexpression of LC3 here is an artificial system, the resulted overexpression of fibronectin may mimic the activation of hepatic stellate cells in response to liver damage. Hepatic stellate cells become activated to enhance the production of cellular fibronectin while the clearance of cellular fibronectin by hepatocytes is impaired so that more fibronectins are accumulated, which eventually leads to liver fibrosis and regeneration (Zhang & Friedman, 2012). As we previously reported, autophagy is required to be enhanced because of the high metabolic stress induced by genome instability in tumor foci (Xie *et al*., 2011b; Jiang *et al*., 2015). In response to the high requirement of autophagy activity, MAP1S is elevated in such tumor foci (Xie *et al*., 2011b; Jiang *et al*., 2015). If tumor cells are not capable of providing sufficient MAP1S to promote autophagy flux to meet the demand, they may induce pyroptosis to impair the survival of themselves and their host cells. It is possibly the reason that prostate cancer patients with lower levels of MAP1S in their tumor tissues had a shorter time of survival than the patients with high levels of MAP1S (Jiang *et al*., 2015).

## Experimental procedures

### Antibodies, plasmids, and other reagents

Monoclonal antibody against MAP1S (Cat# AG10006) was a gift from Precision Antibody^™^, A&G Pharmaceutical, Inc., Columbia, Maryland. Primary antibodies against GFP (SC‐8334), β‐actin (SC‐47778), P27 (SC‐776), EEA1 (SC‐33585), Beclin 1 (SC‐11427), phosphorylated Bcl‐2 (p‐Bcl2, SC‐377576), phosphorylated p27 (p‐p27, SC‐130603), and ATG5 (SC‐33210) were purchased from Santa Cruz Biotechnology, Inc., Dallas, Texas, USA. Antibody against human LC3 (NB 100‐2331) was from Novus Biologicals. Antibodies against α‐SMA (ab‐5694), fibronectin (ab2413), LAMP2 (ab18528), and TGF‐β (ab66043) were from abcam, Cambridge, Massachusetts, USA. Antibodies against γ‐H2AX (9718 S), Bcl‐2 (#2870), PI3KCIII (#4263), H2AX (#2595), and ATG4B (#5299) were from Cell Signaling Technology, Danvers, Massachusetts, USA. Antibody against p62 (BML‐PW9860) was from Enzo Life Sciences International Plymouth Meeting, Pennsylvania, USA. Horseradish peroxidase‐conjugated secondary antibodies against mouse (#172‐1011) and rabbit (#172‐1019) were from Bio‐Rad, Hercules, California, USA. Rhodamine Red‐X goat anti‐mouse IgG (R6393) and FITC rabbit anti‐mouse IgG (A21202) were from Invitrogen, Carlsbad, California, USA. RFP‐LC3 was a gift from Dr. Mizushima (Mizushima *et al*., 2004). Bafilomycin A1, Sirius Red (Direct Red 80, 365548) and Hydroxyproline Assay Kit (MAK008‐1KT) were from Sigma, St. Louis, Missouri, USA.

### Animal experimentation

Animal protocols were approved by the Institutional Animal Care and Use Committee, Institute of Biosciences and Technology, Texas A&M Health Science Center. All animals received humane care according to the criteria outlined in the ‘Guide for the Care and Use of Laboratory Animals’ prepared by the National Academy of Sciences and published by the National Institutes of Health (NIH publication 86‐23 revised 1985). GFP‐LC3 transgenic C57BL/6 mice as described (Mizushima *et al*., 2004) were maintained to carrying single copy of GFP‐LC3 by continuous backcrossing with mice expressing no GFP‐LC3, and then crossed with C57BL/6 wild‐type and MAP1S^−/−^ mice to generate wild type (MAP1S^+/+^:GFP‐LC3^0/0^), GFP‐LC3 transgenic (MAP1S^+/+^:GFP‐LC3^+/0^), MAP1S knockout (MAP1S^−/−^:GFP‐LC3^0/0^), and GFP‐LC3 transgenic MAP1S knockout mice (MAP1S^−/−^:GFP‐LC3^+/0^) as described in detail in our previous publication (Xie *et al*., 2011a). Transgenic expression of GFP‐LC3 and MAP1S deletion occurred in all tissues. Mice were observed to record their survival times when they were found dead or when they were found to be moribund. Survival times for male and female mice were recorded and analyzed for similarity between different sexes. Because no difference was found between different genders of mice with the same genotypes, survival data of male and female mice were combined for further survival analyses. The overall survival and median survival were analyzed by the Kaplan–Meier method. Cox proportional hazard analysis with univariate or multivariate method was used to explore the effect of variables on overall survival. Mice at different ages were weighted and sacrificed. Liver tissues were weighted and frozen or fixed. Fixed samples were embedded in paraffin and sectioned. The body weight, liver weight, and the ratio of liver weight to body weight were recorded. The SAS software was used for all statistical analyses, and a *P* value of <0.05 was considered significant. All statistical analyses were carried out similarly as previously described (Jiang *et al*., 2014).

### Cell culture, histological analysis, immunoblot, and fluorescent confocal microscopy

Liver tissue sections were stained with Hematoxylin and Eosin (H&E) as previously described (Xie *et al*., 2011b). The area occupied by sinusoids was quantified using the NIH software ImageJ. The percentage of sinusoidal space relative to the total area in three fields from three mice was used to indicate the relative intensity of sinusoidal dilation. For detection of liver fibrosis, tissues sections were stained with Sirius Red (Kumar *et al*., 2014). To further quantify the intensity of liver fibrosis, the concentrations of collagen specific amino acid hydroxyproline in liver tissues from 12‐month‐old male mouse littermates were determined with the Hydroxyproline Assay Kit as instructed by the associated manual. Lysates from liver tissues collected from mice at different ages or from cultured cells were prepared and analyzed by immunoblotting as described (Zou *et al*., 2013, 2014). Immortalized MEFs were developed from wild‐type and MAP1S^−/−^ mice (Xie *et al*., 2011a), and cultured in the same way as we described before (Xie *et al*., 2011a; Yue *et al*., 2015). To examine the impact of MAP1S on autophagy flux, MEFs were cultured in the absence or presence of 10 nm bafilomycin A1 for 6 h. The LC3‐II levels were adjusted by their respective levels of β‐actin, and the increased levels of LC3‐II after bafilomycin A1 treatment were designated as the values of autophagy flux according to the established guidelines (Klionsky *et al*., 2012). MEFs cultured in the presence of FITC‐conjugated fibronectin protein were washed. Tissue sections or culture cells on slides were stained with antibody against fibronectin or other antibody and visualized with fluorescent‐conjugated secondary antibody under a Zeiss LSM 510 confocal microscope as described (Zou *et al*., 2013, 2014).

### Isolation of liver nuclei and flow cytometric analysis

Male mice were sacrificed by cervical dislocation. Their liver tissues were rapidly removed and frozen in the liquid nitrogen. The frozen livers were thawed and used to isolate nuclei for flow cytometric analysis as described previously (Xie *et al*., 2011b). The number of cells were plotted against the DNA contents and percentages of cells with DNA contents of ≤2N (G0/G1), 2N‐4N (S), 4N (G2/M) or >4N were calculated for comparison among mice of different genotypes.

### Analysis of oxidative stress

A part of frozen samples was cryosectioned and used for measurement of oxidative stress. The cryosections were stained with 2 mm dihydroethidine hydrochloride for 30 min at 37 °C. Dihydroethidium labels cytosolic superoxide that intercalates into genomic DNA upon oxidation so that nuclei are labeled with red fluorescent signals. The levels of oxidative stress in the cells were monitored by fluorescence microscopy and quantified by the relative levels of red fluorescent intensity in nuclei by image analysis software ImageJ.

## Funding

This work was supported by a NCI R01CA142862 to Leyuan Liu, the National Natural Science Foundation of China (No: 81472382), the Guangdong Province Natural Science Foundation (No: 2014A030313079), the Fundamental Research Funds for the Central Universities (No: 14ykpy19), Guangdong Province Science and Technology for Social Development Project (No: 2013B021800107), and Guangzhou City 2015 Scientific Research Projects (7415600066401) to Hai Huang. The funders had no role in study design, data collection and analysis, decision to publish, or preparation of the manuscript.

## Conflict of interests

The authors declare no competing financial interests.

## Author contributions

W.L. J.Z. F.Y. J.Y. L.L. designed the study, performed most of experiments, and wrote the manuscript. W.L. J.Z. F.Y. K.S. Q.C. G.X. H.H. L.L. performed other experiments. W.L.M. F.W. provided critical reagents. All authors reviewed the results and approved the final version of the manuscript.
